# Safety Evaluation of the Combination with Dexrazoxane and Anthracyclines: A Disproportionality Analysis Based on the Food and Drug Administration Adverse Event Reporting System Database

**DOI:** 10.3390/ph17121739

**Published:** 2024-12-23

**Authors:** Danyi Liu, Junting Liu, Rui Xiao, Anqi Deng, Wei Liu

**Affiliations:** School of Pharmaceutical Sciences, Zhengzhou University, Zhengzhou 450001, China; yxy_liudanyi@gs.zzu.edu.cn (D.L.); adamwell@gs.zzu.edu.cn (J.L.); xiaoyyy@gs.zzu.edu.cn (R.X.); daqemail@gs.zzu.edu.cn (A.D.)

**Keywords:** dexrazoxane, anthracyclines, cardiotoxicity, infection, children, disproportionality analysis

## Abstract

**Objectives**: As one of the important interventions to alleviate anthracycline-related cardiotoxicity (ARC), the safety assessment of dexrazoxane in clinical practice is particularly important. This study aims to evaluate the actual efficacy and potential adverse effects of dexrazoxane in clinical practice by analyzing the reports of adverse events (AEs) related to the combination with dexrazoxane and anthracyclines. **Methods**: We utilized four disproportionality analysis methods to analyze AE reports of the combination with dexrazoxane and anthracyclines in the Food and Drug Administration Adverse Event Reporting System (FAERS) database from the third quarter of 2014 to the first quarter of 2024. **Results**: Under the three backgrounds, a large number of preferred terms (PTs) such as cardiac failure disappeared in the combined group, and the PTs with significant signal values were mainly concentrated in infections and infestations. For patients under 18, some PTs associated with infections and infestations disappeared after the combination of the two drugs. **Conclusions**: Dexrazoxane can effectively alleviate ARC, but it may also increase the risk of infection. For infections and infestations, children under 18 years old are more likely to benefit from the combination therapy. More attention should be paid to infectious AEs in the clinical use of dexrazoxane, though disproportionality analysis is a hypothesis-generating approach.

## 1. Introduction

Anthracyclines are a class of antitumor drugs containing an anthracene structure, which mainly inhibits the growth and division of cancer cells by interfering with the synthesis and function of DNA [1]. The most representative drugs in this class include doxorubicin, daunorubicin, epirubicin, etc. [2,3]. Since anthracyclines were first introduced, they have become an important means of treating breast cancer, leukemia, lymphoma, and other cancers. However, the powerful antitumor activity of anthracyclines is accompanied by several toxic side effects, such as cardiotoxicity, myelosuppression, and mucosal inflammation [4,5,6], which limits the clinical application and dosage of anthracyclines to a certain extent. Anthracycline-related cardiotoxicity (ARC) is a multifactorial process with an unclear mechanism. Anthracyclines have been proven to be able to exert various effects due to oxidative and nitrosative stress in the heart and blood vessels, mitochondrial dysfunction, and cell apoptosis, leading to cardiotoxicity [7,8]. ARC can manifest as asymptomatic cardiac dysfunction, arrhythmia, pericarditis, cardiac valve disease, or myocardial ischemia and may evolve into cardiac failure congestive [9]. Therefore, how to ensure the antitumor efficacy of anthracyclines while reducing their toxic side effects has always been a hot topic in the medical community. Various cardioprotective strategies, including prolonging the infusion time of anthracyclines, using liposomal anthracycline substitution therapy [10,11], and combining the use of neurohormone antagonists and statin drugs [3], have been explored to alleviate ARC.

As the only Food and Drug Administration (FDA)-approved protective agent that can effectively prevent ARC, dexrazoxane is commonly used for the management and treatment of cardiotoxicity and extravasation injury caused by anthracyclines [12] in clinical practice. The mechanism through which dexrazoxane exerts its cardioprotective activity is not fully comprehended. Existing experimental evidence indicates that the ethylenediaminetetraacetic acid (EDTA) form of dexrazoxane can interfere with iron-mediated free reactive oxygen species, which is believed to partly contribute to the development of ARC. Some also hold the view that it is possible for dexrazoxane to have a protective effect by inhibiting the ability of topoisomerase II [13,14]. A retrospective study by Schuler M.K. et al. [15] found that only 7% of patients receiving anthracyclines combined with dexrazoxane experienced ARC during treatment or follow-up. Patients were able to tolerate higher cumulative doses (a median cumulative dose of 750 mg/m^2^) of anthracyclines combined with dexrazoxane, and the combined therapy resulted in a median overall survival of 17 months and a median progression-free survival of 9 months. Additionally, dexrazoxane is a well-tolerated drug, with tolerability in adult cancer patients receiving anthracycline therapy similar to that of the placebo [13]. Reichardt P. [16] et al., through a meta-analysis, found that there was no significant correlation between the drug and excess mortality rate, and it did not impair the antitumor therapeutic efficacy of anthracyclines. Currently, dexrazoxane has been recommended by many international authoritative guidelines to alleviate ARC, such as the ASCO 2008 Clinical Practice Guideline Update: Use of Chemotherapy and Radiation Therapy Protectants [17], the 2022 ESC Guidelines on cardio-oncology [3], the 2023 CSCO Clinical guidelines for oncology cardiology [18,19], and the NCCN Guidelines: B-Cell Lymphomas (Version 3.2024) [20].

Different countries and regions have different clinical guidelines and practices, and there are also differences in the recommendations for the use of dexrazoxane with anthracyclines. For example, the European Union (EU) and the United States (US) have different attitudes towards the use of dexrazoxane in children. In 2011, the European Medicines Agency (EMA) evaluated that the safety and effectiveness of dexrazoxane in children had not been established and that dexrazoxane should not be used in children due to the risk of Second Malignant Neoplasm (SMN) and potentially negative pharmacodynamic interactions with anthracyclines [21]. Therefore, the EMA recommended contraindicating the use of dexrazoxane in children and adolescents under the age of 18. In 2015, the discussion on the prohibition of using dexrazoxane in children and adolescents was submitted to the Committee for Medicinal Products for Human Use (CHMP) for arbitration. CHMP recommended that the contraindication for dexrazoxane should be limited to children and adolescents under the age of 18 who were expected to receive a cumulative dose < 300 mg/m^2^ of doxorubicin or an equivalent cumulative dose of another anthracycline drug. The EMA implemented the CHMP recommendation in the EU on 19 July 2017 [16]. In contrast, in the US, dexrazoxane was designated as an orphan drug by the FDA for the prevention of cardiomyopathy in children and adolescents aged 0–16 who were being treated with anthracyclines in August 2014 [22]. However, in November 2020, the FDA-updated drug label [23] of dexrazoxane in pediatric use was still “the safety and effectiveness have not been established”. A retrospective cohort study [24] investigating the pattern of dexrazoxane usage in pediatric patients with acute lymphoblastic leukemia (ALL) or acute myeloid leukemia (AML) in the Pediatric Health Information Systems database in the US indicated that dexrazoxane administration was limited, and prescribing practices varied widely within and between institutions. The controversies surrounding dexrazoxane also focus on its cardioprotective effects, the potential increase in the risk of SMN, the complexity of monitoring, and the uncertainty of the long-term effects. Two recent comments [25,26] in the *Journal of the American College of Cardiology* have also shown a keen interest in the safety of dexrazoxane in clinical use.

As a spontaneous reporting system, the Food and Drug Administration Adverse Event Reporting System (FAERS) database is a commonly used tool in the field of pharmacovigilance. It is widely used and has played an important role in drug safety monitoring because of its highly efficient and reliable “safety signals” in identifying and evaluating [27,28]. Furthermore, a disproportionality analysis can assist in identifying potential association signals between drug use and adverse reactions. Zeming Mo [29] et al. collected and analyzed FAERS data from January 2004 to June 2022 to draw the conclusion that children under 18 were more likely to benefit from dexrazoxane in preventing cardiac failure, but the study did not use a disproportionality analysis to detect dexrazoxane signals in the FAERS database, compare the differences in adverse events (AEs) between children under 18 and adults over 18 who used dexrazoxane, or analyze the new clinical risks associated with the combination of dexrazoxane and anthracyclines.

Therefore, this study intends to use a disproportionality analysis to detect the signals of AEs of dexrazoxane combined with anthracyclines in the FAERS database under different background datasets, further explore the benefits and risks of the age factor in the combination of these two drugs, and reveal the actual effect and potential AEs of dexrazoxane in alleviating ARC in the real world, so as to provide more solid and reliable data support for clinical decision-making.

## 2. Results

### 2.1. Basic Clinical Features of the Reports

Among the 60,199 reports, 59,489 were in the anthracycline group, 597 were in the combined group, and only 113 were in the dexrazoxane alone group. The basic clinical features of the reports are summarized in Table 1. The proportion of male and female patients in the three groups was basically even, close to 1:1. In terms of age distribution, the patients in the anthracycline group were mainly aged 45–64 years old and 65 years old and above. In contrast, the age range of patients in the combined group and the dexrazoxane alone group was biased towards < 18 years old. The reporting countries of the anthracycline group were widely distributed, and not limited to the US. The reporting countries of the combined group were mainly the US and Canada, while the dexrazoxane alone group was mainly reported in Japan, accounting for 40.71%. In terms of reported outcomes, all three groups focused on hospitalization (initial or prolonged) and other serious outcomes (important medical events). In addition, the reporters of all three groups were mainly from healthcare professionals, highlighting the central role of healthcare professionals in AE monitoring and reporting.

### 2.2. AE Signal Analysis of the Three Groups in Three Different Backgrounds

We extracted top 30 preferred terms (PTs) from each of the three groups and removed PTs unrelated to the drug, such as off label use, product use issue, and drug ineffective. A total of 47 PTs (including one indication PT—acute myeloid leukaemia) were integrated, involving 13 system organ class (SOC) systems. Table 2 shows the results of the signal detection with four methods for 47 PTs based on the background of all datasets.

Under the background of all datasets, the combined group had 25 PTs detected by four methods, while 10 PTs, such as thrombocytopenia, vomiting, sepsis, and hypotension, were not detected by the Medications and Health Care Products Regulatory Agency (MHRA). Additionally, 12 PTs including leukopenia, anaemia, asthenia, and dyspnoea, were not detected by any method.

**Figure 1:**
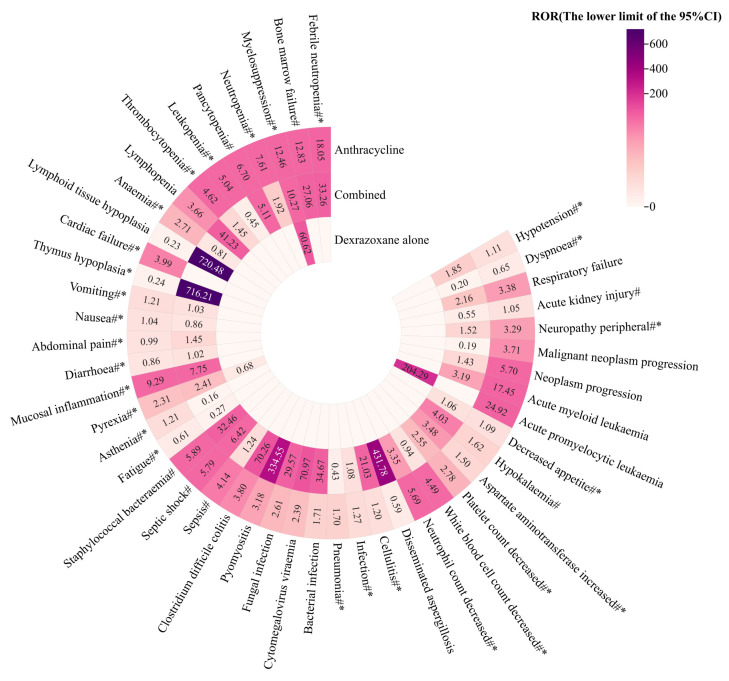
Comparison of AE signal detection results for the three groups under the background of all datasets. #: PT in anthracyclines labels; *: PT in dexrazoxane labels.

Based on the 47 PTs, combined with Figure 1 and Table 2, under the background of all datasets, no positive signals were found in the anthracycline group, while there were five positive signals in the combined group, including lymphoid tissue hypoplasia (ROR _the lower limit of 95%CI_: 720.48), thymus hypoplasia (ROR _the lower limit of 95%CI_: 716.21), abdominal pain (ROR _the lower limit of 95%CI_: 1.45), diarrhoea (ROR _the lower limit of 95%CI_: 1.02), and disseminated aspergillosis (ROR _the lower limit of 95%CI_: 431.78). Among them, the reporting numbers of lymphoid tissue hypoplasia, thymus hypoplasia, and disseminated aspergillosis exceeded 50, with the ROR lower limit of 95%CI being more than 400, significantly higher than that for other PTs. None of the above three PTs were mentioned in the EU and the US labels of anthracyclines and dexrazoxane, suggesting that the combination of these two drugs may cause other potential risks in clinical practice.

There were 15 PTs with signals in the anthracycline group, which were enhanced in the combined group, including febrile neutropenia, septic shock, pyrexia, hypotension, etc., involving six SOC systems, most of which were concentrated in infections and infestations (8 PTs) and blood and lymphatic system disorders (3 PTs). The ROR lower limit of 95%CI of pyomyositis in the combined group was as high as 334.55, which was 105 times that in the anthracycline group. Other PTs related to infections and infestations such as staphylococcal bacteraemia (ROR _the lower limit of 95%CI_: 32.46), cytomegalovirus viraemia (ROR _the lower limit of 95%CI_: 70.97), bacterial infection (ROR _the lower limit of 95%CI_: 34.67), and fungal infection (ROR _the lower limit of 95%CI_: 29.57) also showed strong signals, far exceeding the signal level detected by the anthracycline group.

There were 23 PTs with signals in the anthracycline group, which decreased in the combined group. The signals of cardiac failure and acute promyelocytic leukaemiadisappeared completely after the combination of dexrazoxane and anthracyclines, which further confirmed the potential protective effect of dexrazoxane in alleviating ARC.

**Figure 2:**
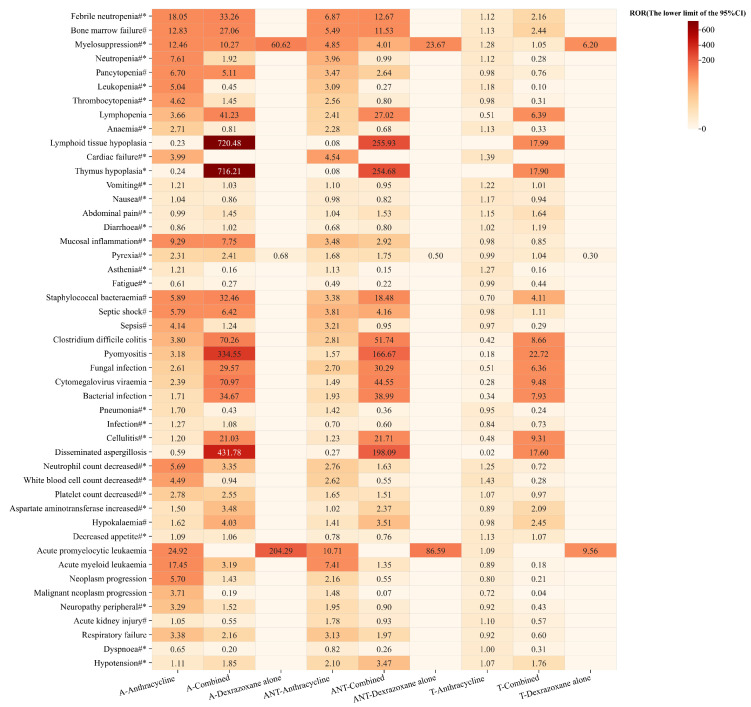
Comparison of AE signal detection results for the three groups under three backgrounds. #: PT in anthracyclines labels; *: PT in dexrazoxane labels.

AE signals were detected separately under the background of all datasets, antitumor data subsets, and target data subsets, and the results are shown in Figure 2, with the darker color indicating larger ROR signal values. Under the background of all datasets, the PTs with larger ROR signal values in the anthracycline group were concentrated in blood and lymphatic system disorders; investigations; and neoplasms benign, malignant and unspecified (incl cysts and polyps). The situation was different for the combined group, and the PTs with larger ROR signal values were concentrated in blood and lymphatic system disorders, and infections and infestations.

As the background datasets shrink, the signal intensity of the three groups showed an overall decreasing trend, and the differences in PTs between the three groups gradually became prominent. Compared with the background of all datasets, under the background of antitumor data subsets, the signal values of PTs in the combined group related to blood and lymphatic system disorders were significantly reduced, and the PTs were more concentrated in infections and infestations, among which the signal values of fungal infection (ROR _the lower limit of 95%CI_: 30.29), bacterial infection (ROR _the lower limit of 95%CI_: 38.99), and cellulitis (ROR _the lower limit of 95%CI_: 21.71) did not decrease but increased. Under the target data subsets, the differences between the combined group and the anthracycline group were more obvious, especially in infections and infestations, with more prominent signal values of PTs.

Based on the comparison of the above signals, it can be seen that infections and infestations are the most prominent risks of the combined group, especially for nine PTs, including staphylococcal bacteraemia, clostridium difficile colitis, pyomyositis, etc., which produced strong positive signals under the three backgrounds. In addition, individual PTs such as febrile neutropenia, bone marrow failure, lymphopenia, lymphoid tissue hypoplasia, thymus hypoplasia, aspartate aminotransferase increased, hypotension, etc. also had relatively clear signals, and the ROR lower limit of 95%CI was always > 2 under the three backgrounds.

### 2.3. Analysis of AE Signals in Cardiac Disorders

**Figure 3:**
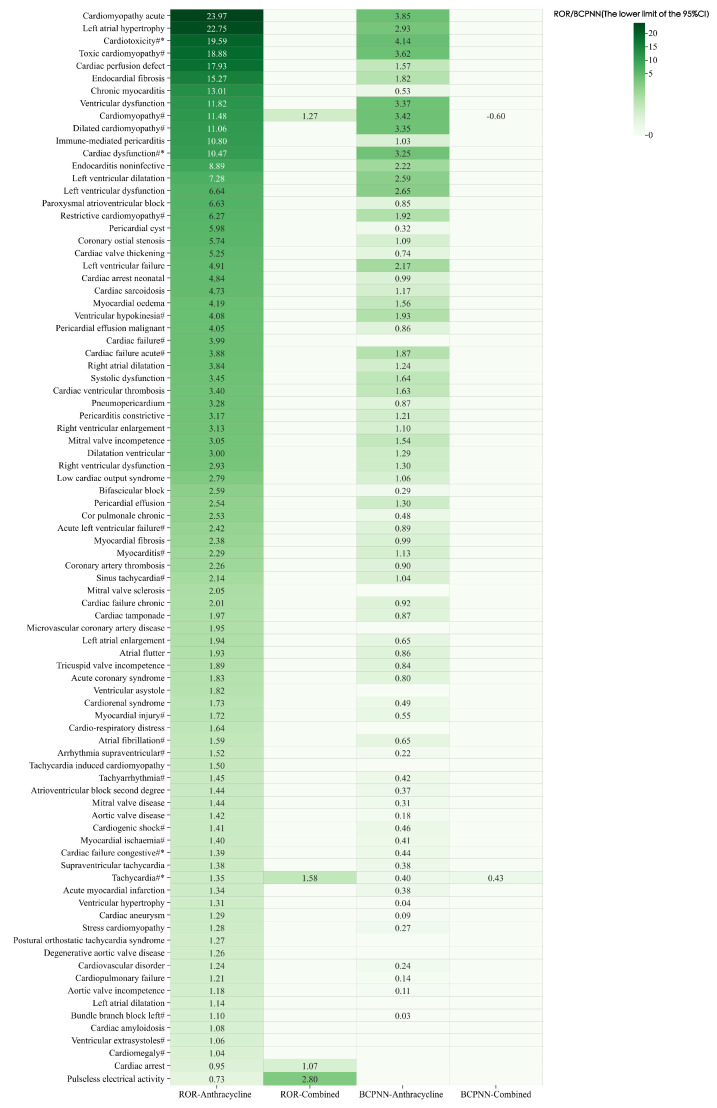
Comparison of AE signal detection results for the two groups with cardiac disorders under the background of all datasets. #: PT in anthracyclines labels; *: PT in dexrazoxane labels.

Under the background of all datasets, the dexrazoxane alone group did not detect any positive signals for cardiac disorders, while the remaining two groups detected a total of 136 PTs related to cardiac disorders. The ROR and BCPNN signal detection results are shown in Figure 3 after excluding PTs with no positive signals in both groups.

The PT values with higher anthracycline signal intensity are mainly concentrated in cardiomyopathy. Cardiotoxicity (ROR _the lower limit of 95%CI_: 19.59, BCPNN _the lower limit of 95%CI_: 4.14), cardiomyopathy acute (ROR _the lower limit of 95%CI_: 23.97, BCPNN _the lower limit of 95%CI_: 3.85), and toxic cardiomyopathy (ROR _the lower limit of 95%CI_: 18.88, BCPNN _the lower limit of 95%CI_: 3.62) ranked high in the anthracycline group using the BCPNN method. Compared with the anthracycline group, PTs detected with cardiac disorders were significantly reduced in the combined group. The signal intensity of cardiomyopathy (ROR _the lower limit of 95%CI_: 1.27, BCPNN _the lower limit of 95%CI_: −0.60) was significantly reduced in the combined group, indicating that dexrazoxane can indeed alleviate ARC.

### 2.4. Analysis of AE Signals in Age Subgroups

**Figure 4:**
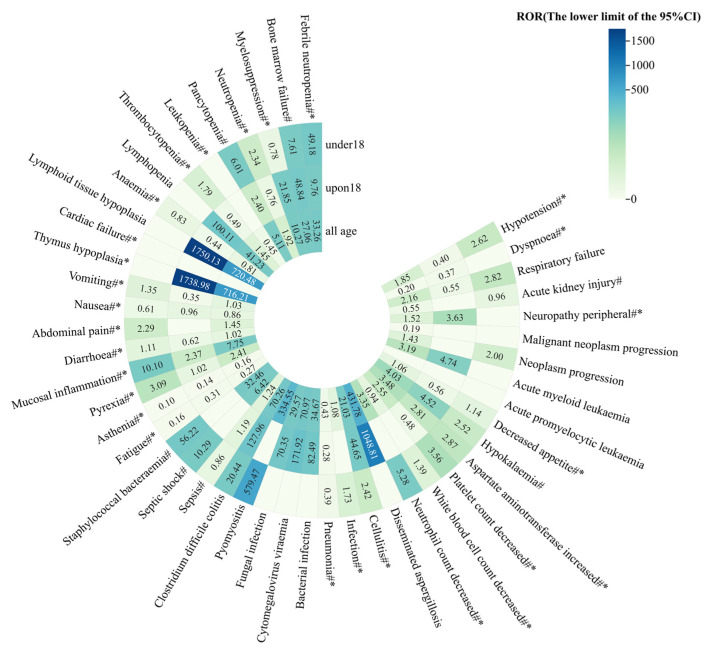
Comparison of AE signal detection results for age subgroups under the background of all datasets. #: PT in anthracyclines labels; *: PT in dexrazoxane labels.

We stratified the reports of the combined group by age and divided them into two subgroups: under 18 (median age: 7 years old, average age: 7.71 years old) and over 18 (median age: 52 years old, average age: 53.52 years old). Signal detecting was performed under the background of all datasets, and the results are shown in Figure 4.

Based on the 47 PTs mentioned previously, the distribution of positive signals in the over 18 group was basically the same as that in the all age group. PTs such as lymphoid tissue hypoplasia (ROR _the lower limit of 95%CI_: 1750.13), thymus hypoplasia (ROR _the lower limit of 95%CI_: 1738.98), and disseminated aspergillosis (ROR _the lower limit of 95%CI_: 1048.81) showed strong signals in the over 18 group and a ROR lower limit of 95%CI > 1000, which was more than twice that of the all age group. In the under 18 group, only the ROR signal values of pyomyositis was more prominent, with the lower limit of 95%CI reaching 579.47. For infections and infestations, the combination of dexrazoxane and anthracyclines was more advantageous in the under 18 group. The number of signals for sepsis, clostridium difficile colitis, pneumonia, and cellulitis decreased, and signals for fungal infection, cytomegalovirus viraemia, bacterial infection, and disseminated aspergillosis disappeared.

## 3. Discussion

### 3.1. Dexrazoxane Can Effectively Alleviate ARC

For ARC such as cardiomyopathy, cardiac failure, cardiac failure congestive, acute left ventricular failure, arrhythmia, electrocardiogram change, heart injury, and others, the treatment regimen combining dexrazoxane with anthracyclines showed beneficial signal results, and a large number of cardiac-related AEs disappeared, proving that the regimen combined with dexrazoxane can play a role in alleviating ARC.

Dexrazoxane is an EDTA cyclic derivative that can penetrate cell membranes [14,30]. It is evident that dexrazoxane can be converted into an open-ring chelator within cells, which can chelate with free iron to interfere with iron-mediated free radical generation, reduce the production of anthracycline–metal complexes, and reduce the content of lipid peroxidation products. Additionally, dexrazoxane can also inhibit topoisomerase II to interfere with DNA regulation, thereby reducing cardiomyocyte apoptosis and providing protection [30,31,32].

The prospective study by Sánchez-Medina J et al. [33] concluded that the use of dexrazoxane from the beginning of treatment was effective in preventing clinically symptomatic cardiac failure. Dexrazoxane allowed high doses and intensities of anthracyclines to be administered in children with AML and to exceed the recommended maximum dose of anthracyclines without subclinical cardiac injury (evidenced by the left ventricular ejection fraction (LVEF) on echocardiography). In a pediatric trial (P9754) [34,35] involving 242 patients with bone sarcoma, all patients received doxorubicin (cumulative dose between 450 and 600 mg/m^2^) combined with dexrazoxane. Only five patients had a 50% decrease in LVEF, four of which were transient, and there were no cardiac failure events. The concomitant use of dexrazoxane allowed for a cumulative dose of doxorubicin up to 600 mg/m^2^. A recent Cochrane review [36], based on five RCTs (three in adults and two in children), reported significant differences between the combined treatment groups for clinical cardiac failure and subclinical cardiac dysfunction (risk ratio 0.24–0.56 in adults; risk ratio 0.33–0.66 in children), with beneficial results for dexrazoxane. Multiple meta-analysis results [37,38] have shown that dexrazoxane can reduce the risk of clinical cardiac failure and cardiac AEs in patients receiving anthracycline chemotherapy, without reducing the response rate of antitumor drugs, and has no significant effect on cancer prognosis.

A large body of evidence has shown the efficacy of dexrazoxane in alleviating ARC. However, according to the number of adverse reaction reports, the combination of dexrazoxane and anthracyclines is not frequently used. This phenomenon may be attributed to the safety profiles of these two drugs; however, it also reflects a disconnect between clinical trial evidence and real-world clinical practice.

### 3.2. Dexrazoxane May Increase the Patient’s Risk of Infection

Signal detection results in the three backgrounds showed that the combination of dexrazoxane and anthracyclines may increase the incidence of some non-cardiotoxicity AEs, like infections and infestations, such as staphylococcal bacteraemia, pyomyositis, disseminated aspergillosis, and others, and blood and lymphatic system disorders, such as febrile neutropenia and bone marrow failure.

The results of signal detection under the three backgrounds suggested the occurrence of infection from multiple perspectives. Myelosuppression and bone marrow failure may be associated with moderate to severe life-threatening complications and may further increase the patient’s risk of serious infections [39]. Regarding myelosuppression, a significant signal was detected when dexrazoxane was used alone, indicating that dexrazoxane did have some hematotoxicity side effects and a risk of causing myelosuppression. However, the ROR signal value of myelosuppression in the combined group was lower than that in the dexrazoxane alone group or anthracycline group, which reflected the advantages of dexrazoxane for patients receiving anthracyclines. At the same time, lymphoid tissue hypoplasia, thymus hypoplasia, and lymphopenia with prominent ROR signal values also reflected strong depletion of lymphocytes and the body’s immune system, and such phenomena had also been confirmed in various infections such as AIDS, experimental bacterial infection, and fungal infection [40,41]. The occurrence of AEs such as febrile neutropenia and pyrexia further suggested that the patient may be in a certain state of infection.

An earlier review [13] reported that dexrazoxane was associated with a higher incidence of leukopenia (78% vs. 68%; *p* < 0.01), while white blood cells play an important role in the body’s immune system defense against infection and cancer. The use of dexrazoxane easily causes the patient’s immune function to be compromised by pathogenic microorganisms such as fungi, bacteria, and viruses, which leads to infections and infestations, resulting in an increase in treatment costs, the use of antibiotics, the prolongation of hospital stay, a reduction in or delay of chemical drugs, and severe cases. It can also lead to life-threatening complications such as septic shock, sepsis syndrome, and even death of the patient [42].

Therefore, correct evaluation and use of dexrazoxane, early identification of the risk of bone marrow suppression and various infections, and reasonable prevention and treatment are of great significance to reduce the complications related to dexrazoxane combined with anthracyclines and to improve the safety of patients and the efficacy of antitumor chemotherapy.

### 3.3. The Use-of-Dexrazoxan Trend Was Age-Related

According to the above signal detection results, the use of dexrazoxane greatly reduces the occurrence of ARC, so there are fewer AEs of cardiac disorders in all age groups. It is difficult to accurately evaluate and compare the benefits and risks of dexrazoxane treatment in different age groups. However, for the risk of infections and infestations revealed in this study, the signal detection results of age subgroups showed that children under 18 were more likely to benefit from the combination regimen. Adolescent cancer patients are more likely to benefit from dexrazoxane in infections and infestations, possibly due to differences in the immune response between children and adults [43]. The innate immune response (an early response to a wide range of pathogens) is often more active in children. Compared with the adaptive immune response in adults, the innate immune response is faster and more effective. With aging, innate lymphocytes in the blood decrease, and the immune killing function of the cells decreases [44,45]. Therefore, for elderly cancer patients, the risk of adverse reactions such as infections are also related to many factors such as aging, comorbidities, and poor lifestyle choices.

It is suggested that the reason why dexrazoxane is not widely used in patients under 18 may be due to its potential risk of SMN [46]. Compared with the anthracycline group, the results of signal detection showed that under the background of all datasets and antitumor data subsets, the combined group produced lower positive signal intensity in PTs of neoplasms benign, malignant and unspecified (incl cysts and polyps). Under the background of the target data subsets, the combined group did not detect positive signals. The Dana-Farber Cancer Institute ALL Consortium Protocol 95-01 [47] found that dexrazoxane was not associated with an increased risk of SMN with a median follow-up of 6.2 years. Seif AE et al. [48] conducted a retrospective analysis of 15,532 cancer patients (30% <5 years old; 70% >3 to <15 years old) from 43 children’s hospitals. Of these, 1406 received dexrazoxane as a cardioprotective agent, and no association was found between dexrazoxane and the risk of SMN (incidence of secondary AML dexrazoxane vs. non-dexrazoxane was 0.21% vs. 0.55%). Furthermore, dexrazoxane was not significantly associated with secondary AML in adjusted models that included etoposide. The Cochrane analysis by de Baat EC et al. [36] confirmed that dexrazoxane can reduce the risk of cardiac failure (2.5–4.5-fold compared with the placebo) without compromising the antitumor efficacy of chemotherapy or increasing the risk of SMN in adults. At the same time, relevant studies also pointed out that the underlying disease and chemotherapy itself can make patients prone to SMN, especially when patients are treated with a variety of cytotoxic drugs.

## 4. Materials and Methods

### 4.1. Data Sources

The research data were sourced from the FAERS database (https://fis.fda.gov/extensions/FPD-QDE-FAERS/FPD-QDE-FAERS.html) (accessed on 1 June 2024). After eliminating duplicate reports and those with ‘NA’ content, we extracted a total of 7,343,712 reports of drugs with roles as the primary suspect, as the secondary suspect, and in the interaction (PS, SS, I) from 39 quarters (2014 Q3–2024 Q1) of the FAERS database for all datasets. To reduce the interference of related disease backgrounds and further compare the AE characteristics of the two drugs, we extracted reports of antitumor drugs and target drugs as two analysis data subsets. The background/group settings and report numbers are shown in Table 3. The selection of antitumor drugs was based on the active ingredients corresponding to the Anatomical Therapeutic Chemical (ATC) [49] classification system with group L01, and anthracyclines were group L01DB. It is important to note that several different antitumor drugs or anthracyclines may have been reported in a single report, and all of them were included in the statistics. Meanwhile, the specific salts of anthracyclines were also regarded as anthracyclines. Considering that the use of liposomal anthracyclines was also a protective strategy, the reports of liposomal anthracyclines were excluded in order to more purely explore the utility of dexrazoxane in alleviating ARC. So, the anthracyclines studied in this paper refer to anthracyclines in common dosage forms.

### 4.2. AE Determination

AEs were coded and classified using vision 27.0 Medical Dictionary for Regulatory Activities (MedDRA) SOC systems and PTs.

### 4.3. Statistical Methods

In this study, a disproportionality analysis was used to detect AE signals, including the reporting odds ratio (ROR), the proportional reporting ratio (PRR), the Medications and Health Care Products Regulatory Agency (MHRA), and the Bayesian confidence propagation neural network (BCPNN). The data such as target drug-ADR and non-target drug-ADR are obtained by constructing a 2 × 2 list, and the signal values for each method were calculated according to the formula [50,51]. A positive signal was considered statistically significant between the target drug and the target AE. The conditions under which a positive signal was detected by each method are shown in Table 4 [50,51,52,53,54]. R (4.4.1) software was used for data cleaning, processing, and analysis. Considering that ROR detected more positive signals and the stability and accuracy of the BCPNN method are relatively high, we chose to visualize both the ROR and the BCPNN detection results in the form of a heat map for easy interpretation. However, if the trends and results shown by both methods are consistent, we will only use the ROR detection results, just like in Section 2.2 and Section 2.4 of the Results section.

## 5. Conclusions

Our study provides relevant data support for the safety of dexrazoxane in clinical application. The signal detection results based on the FAERS database can be used to conclude that the risk of anthracycline-related cardiotoxicity, such as cardiac failure, cardiac failure congestive, cardiotoxicity, etc., is lower in patients receiving the combination of dexrazoxane and anthracyclines. Patients treated with dexrazoxane are more likely to suffer from infections and infestations. Compared with adult patients, children under 18 are more likely to benefit from the combination regimen. This suggests that the identification and monitoring of patients’ infection status should be strengthened in clinical practice, and the treatment plan should be modified as soon as possible when the drug damage exceeds its benefits. In the statistical data, we also found seven cases of dexrazoxane combined with liposomal anthracyclines, but the number of reports was small, and signal detection could not be carried out, which also suggested that dexrazoxane combined with liposomal anthracyclines is used in clinical practice, and its benefits and risks need to be further explored. We believe dexrazoxane will play an important role in more areas in the future. At the same time, we also need to pay attention to its potential risks and limitations in order to better guide clinical practice and ensure patient safety.

It should be noted that the reports in the FAERS database may have certain biases because of its spontaneous reporting nature: the quality of the reports varies greatly, and there are also cases of under-reporting. As the data mainly come from the United States, the results’ global applicability still needs further verification. Additionally, the adverse event signals obtained by the disproportionality analysis in this study are based on the quantitative association of adverse reaction reports, rather than a biological association, and do not represent an inevitable causal relationship between the drugs and adverse events. Although the ROR method has the advantages of high sensitivity and eliminating a large amount of bias, the adverse event signals detected by it are greatly affected by the number of adverse reaction reports. More data collection and reporting are still needed in future clinical studies to improve the opinions of experts on drug safety.

Our next step is to detect adverse event signals by using other pharmacovigilance databases, including the Japanese Adverse Drug Event Reporting System, the European Union Drug Regulating Authorities Pharmacovigilance, the WHO VigiBase, and so on. If possible, we will also further assess the efficacy of anthracyclines combined with dexrazoxane using clinical studies and real clinical data from medical institutions, reducing the risk of drug use and ensuring the effectiveness of the patient’s treatment.

## Figures and Tables

**Table 1:** Basic clinical features of the reports.

Characteristic	Anthracycline (N = 59,489)	Combined (N = 597)	Dexrazoxane Alone (N = 113)
Gender, N (%)			
Female	32,714 (54.99)	271 (45.39)	59 (52.21)
Male	26,766 (44.99)	326 (54.61)	54 (47.79)
Unknown	9 (0.02)	0	0
Age (years), N (%)			
<18	8664 (14.56)	369 (61.81)	85 (75.22)
18–44	12,861 (21.62)	99 (16.58)	7 (6.19)
45–64	22,142 (37.22)	63 (10.55)	13 (11.5)
≥65	15,822 (26.6)	66 (11.06)	8 (7.08)
Reported Countries, N (%)			
United States	12,270 (20.63)	214 (35.85)	29 (25.66)
Canada	4068 (6.84)	214 (35.85)	6 (5.31)
France	7100 (11.93)	10 (1.68)	3 (2.65)
Italy	4417 (7.42)	5 (0.84)	1 (0.88)
China	5392 (9.06)	30 (5.03)	2 (1.77)
Japan	4186 (7.04)	62 (10.39)	46 (40.71)
Other Country	21,328 (35.85)	48 (8.04)	16 (14.16)
Unknown	728 (1.22)	14 (2.35)	10 (8.85)
Outcomes, N (%)			
With Outcome	58,638 (98.57)	587 (98.32)	107 (94.69)
Death	8988 (10.98)	79 (8.88)	7 (5.93)
Life-Threatening	6178 (7.55)	62 (6.97)	3 (2.54)
Disability	1011 (1.23)	2 (0.22)	1 (0.85)
Hospitalization—Initial or Prolonged	25,792 (31.5)	442 (49.66)	7 (5.93)
Required Intervention to Prevent Permanent Impairment/Damage	33 (0.04)	0	0
Congenital Anomaly	112 (0.14)	5 (0.56)	0
Other Serious Outcome (Important Medical Event)	39,753 (48.56)	300 (33.71)	100 (84.75)
Reporters, N (%)			
Healthcare Professional	55,072 (92.58)	502 (84.09)	101 (89.38)
Non-Healthcare Professional	3345 (5.62)	39 (6.53)	2 (1.77)
Missing	1072 (1.8)	56 (9.38)	10 (8.85)

**Table 2:** AE signal detection results of four methods based on all datasets.

PT ^1^	SOC ^2^	Anthracycline					Combined					Dexrazoxane Alone				
		Count	ROR ^3,8^	PRR ^4,8^	MHRA ^5^	BCPNN ^6^	Count	ROR	PRR	MHRA	BCPNN	Count	ROR	PRR	MHRA	BCPNN
Febrile neutropenia #*	Blood and lymphatic system disorders	6539	18.05 § ^7^	17.52 §	17.59;97,905.26 §	4.03 §	208	33.26 §	31.36 §	32.03;6992.83 §	4.81 §	/	/	/	/	/
Bone marrow failure #		1331	12.83 §	12.76 §	12.87;14,589.69 §	3.59 §	54	27.06 §	26.72 §	27.88;1737.18 §	4.28 §	/	/	/	/	/
Myelosuppression #*		1766	12.46 §	12.36 §	12.46;18,547.35 §	3.55 §	31	10.27 §	10.21 §	10.81;376.23 §	2.98 §	14	60.62 §	58.67 §	63.64;1240.26 §	3.59 §
Neutropenia #*		4208	7.61 §	7.48 §	7.52;23,909.35 §	2.86 §	24	1.92 §	1.92 §	2.04;27.22 §	0.78 §	/	/	/	/	/
Pancytopenia #		1801	6.70 §	6.65 §	6.70;8962.82 §	2.69 §	29	5.11 §	5.09 §	5.39;151.87 §	2.10 §	/	/	/	/	/
Leukopenia #*		1172	5.04 §	5.02 §	5.07;4023.30 §	2.29 §	4	0.45	0.45	0.52;0.01	−1.54	/	/	/	/	/
Thrombocytopenia #*		2283	4.62 §	4.58 §	4.61;6690.37 §	2.16 §	17	1.45 §	1.45 §	1.57;11.69	0.35 §	/	/	/	/	/
Lymphopenia		277	3.66 §	3.66 §	3.73;640.94 §	1.82 §	54	41.23 §	40.72 §	42.49;2698.80 §	4.71 §	/	/	/	/	/
Anaemia #*		2076	2.71 §	2.69 §	2.71;2403.33 §	1.41 §	15	0.81	0.81	0.88;1.01	−0.46	/	/	/	/	/
Lymphoid tissue hypoplasia		3	0.23	0.23	0.28;0.66	−2.48	55	720.48 §	710.91 §	742.35;47,516.62 §	6.18 §	/	/	/	/	/
Cardiac failure #*	Cardiac disorders	1144	3.99 §	3.97 §	4.01;2753.50 §	1.96 §	/	/	/	/	/	/	/	/	/	/
Thymus hypoplasia *	Congenital, familial, and genetic disorders	3	0.24	0.24	0.28;0.62	−2.46	54	716.21 §	706.90 §	738.46;46,497.07 §	6.15 §	/	/	/	/	/
Vomiting #*	Gastrointestinal disorders	1906	1.21 §	1.20 §	1.21;101.48	0.26 §	33	1.03 §	1.03 §	1.09;4.23	−0.05 §	/	/	/	/	/
Nausea #*		2443	1.04 §	1.03 §	1.04;13.27	0.04 §	40	0.86	0.86	0.91;0.88	−0.30	/	/	/	/	/
Abdominal pain #*		861	0.99	0.99	1.00;2.68	−0.03	26	1.45 §	1.45 §	1.54;14.53	0.40 §	/	/	/	/	/
Diarrhoea #*		1748	0.86	0.86	0.87;17.49	−0.22	40	1.02 §	1.02 §	1.07;3.89	−0.07 §	/	/	/	/	/
Mucosal inflammation #*	General disorders and administration site conditions	1313	9.29 §	9.24 §	9.32;9935.30 §	3.14 §	24	7.75 §	7.72 §	8.23;219.78 §	2.56 §	/	/	/	/	/
Pyrexia #*		3299	2.31 §	2.29 §	2.30;2589.67 §	1.18 §	64	2.41 §	2.39 §	2.48;86.30 §	1.17 §	3	0.68	0.69	0.83;0.84	−1.20
Asthenia #*		1411	1.21 §	1.21 §	1.22;82.37	0.26 §	6	0.16	0.16	0.18;7.50	−2.81	/	/	/	/	/
Fatigue #*		1520	0.61	0.61	0.61;312.19	−0.73	16	0.27	0.28	0.30;11.48	−1.97	/	/	/	/	/
Staphylococcal bacteraemia #	Infections and infestations	148	5.89 §	5.89 §	6.04;725.42 §	2.46 §	17	32.46 §	32.36 §	34.93;798.20 §	3.59 §	/	/	/	/	/
Septic shock #		1361	5.79 §	5.76 §	5.81;5642.68 §	2.48 §	31	6.42 §	6.38 §	6.75;214.34 §	2.41 §	/	/	/	/	/
Sepsis #		1893	4.14 §	4.11 §	4.14;4717.40 §	2.01 §	14	1.24 §	1.24 §	1.35;6.94	0.11 §	/	/	/	/	/
Clostridium difficile colitis		237	3.80 §	3.80 §	3.88;590.69 §	1.87 §	72	70.26 §	69.01 §	71.59;5988.31 §	5.37 §	/	/	/	/	/
Pyomyositis		12	3.18 §	3.18 §	3.49;40.37 §	1.25 §	17	334.55 §	333.56 §	360.42;8346.43 §	4.22 §	/	/	/	/	/
Fungal infection		287	2.61 §	2.60 §	2.65;357.57 §	1.34 §	56	29.57 §	29.19 §	30.43;1968.64 §	4.39 §	/	/	/	/	/
Cytomegalovirus viraemia		102	2.39 §	2.39 §	2.46;123.66 §	1.18 §	49	70.97 §	70.16 §	73.38;4330.50 §	5.11 §	/	/	/	/	/
Bacterial infection		167	1.71 §	1.71 §	1.75;80.32	0.73 §	56	34.67 §	34.22 §	35.68;2325.97 §	4.56 §	/	/	/	/	/
Pneumonia #*		2047	1.70 §	1.69 §	1.70;674.90	0.74 §	13	0.43	0.43	0.47;1.42	−1.35	/	/	/	/	/
Infection #*		820	1.27 §	1.27 §	1.28;77.75	0.33 §	16	1.08 §	1.08 §	1.17;4.61	−0.05 §	/	/	/	/	/
Cellulitis #*		217	1.20 §	1.20 §	1.22;20.93	0.22 §	63	21.03 §	20.72 §	21.56;1517.24 §	4.04 §	/	/	/	/	/
Disseminated aspergillosis		8	0.59	0.59	0.66;0.07	−1.00	55	431.78 §	426.07 §	444.71;28,965.22 §	6.08 §	/	/	/	/	/
Neutrophil count decreased #*	Investigations	978	5.69 §	5.67 §	5.72;4010.98 §	2.46 §	14	3.35 §	3.35 §	3.64;49.05 §	1.38 §	/	/	/	/	/
White blood cell count decreased #*		1534	4.49 §	4.46 §	4.50;4377.29 §	2.12 §	9	0.94	0.94	1.05;2.50	−0.34	/	/	/	/	/
Platelet count decreased #*		933	2.78 §	2.77 §	2.80;1190.18 §	1.44 §	19	2.55 §	2.54 §	2.73;39.49 §	1.12 §	/	/	/	/	/
Aspartate aminotransferase increased #*		315	1.50 §	1.50 §	1.53;84.96	0.55 §	16	3.48 §	3.48 §	3.76;56.95 §	1.47 §	/	/	/	/	/
Hypokalaemia #	Metabolism and nutrition disorders	383	1.62 §	1.62 §	1.65;132.45	0.67 §	20	4.03 §	4.02 §	4.31;82.56 §	1.71 §	/	/	/	/	/
Decreased appetite #*		890	1.09 §	1.09 §	1.10;20.14	0.11 §	19	1.06 §	1.06 §	1.14;4.30	−0.07 §	/	/	/	/	/
Acute promyelocytic leukaemia	Neoplasms benign, malignant and unspecified (incl cysts and polyps)	127	24.92 §	24.90 §	25.65;3147.94 §	4.31 §	/	/	/	/	/	3	204.29 §	204.68 §	245.25;1304.93 §	0.72 §
Acute myeloid leukaemia		1201	17.45 §	17.36 §	17.52;18,408.92 §	4.00 §	7	3.19 §	3.19 §	3.6;28.44 §	0.98 §	/	/	/	/	/
Neoplasm progression		924	5.70 §	5.68 §	5.74;3810.81 §	2.46 §	7	1.43 §	1.43 §	1.61;7.45	0.10 §	/	/	/	/	/
Malignant neoplasm progression		1338	3.71 §	3.69 §	3.72;2839.29 §	1.85 §	3	0.19	0.19	0.22;1.41	−2.78	/	/	/	/	/
Neuropathy peripheral #*	Nervous system disorders	1030	3.29 §	3.27 §	3.31;1797.62 §	1.68 §	12	1.52 §	1.52 §	1.67;11.02	0.35 §	/	/	/	/	/
Acute kidney injury #	Renal and urinary disorders	1108	1.05 §	1.04 §	1.05;11.60	0.05 §	14	0.55	0.55	0.6;0.15	−1.00	/	/	/	/	/
Respiratory failure	Respiratory, thoracic, and mediastinal disorders	993	3.38 §	3.37 §	3.40;1826.11 §	1.72 §	15	2.16 §	2.16 §	2.34;25.35 §	0.85 §	/	/	/	/	/
Dyspnoea #*		1363	0.65	0.65	0.66;192.69	−0.62	11	0.20	0.21	0.23;12.47	−2.41	/	/	/	/	/
Hypotension #*	Vascular disorders	967	1.11 §	1.11 §	1.12;26.49	0.13 §	32	1.85 §	1.84 §	1.95;30.13	0.76 §	/	/	/	/	/

^1^ PT, preferred term. ^2^ SOC, system organ class. ^3^ ROR, reporting odds ratio. ^4^ PRR, the proportional reporting ratio. ^5^ MHRA, the Medications and Health Care Products Regulatory Agency. ^6^ BCPNN, Bayesian confidence propagation neural network. ^7^ §: positive signals; #: PT in anthracyclines labels; *: PT in dexrazoxane labels. ^8^ The ROR/PRR in this table refers to the lower limit of the 95%CI of ROR/PRR.

**Table 3:** Background and group settings.

Background/Group	Setting Method	Number of Reports
All datasets (A-)	drug roles as primary suspect, secondary suspect, and interaction (PS, SS, I) from 39 quarters	7,343,712
Antitumor data subsets (ANT-)	full reports of the active ingredients corresponding to the ATC classification system with group L01	1,172,643
Target data subsets (T-)	reports of the active ingredients corresponding to the ATC classification system with groups L01DB and V03AF02	60,199
Anthracycline	reports of the active ingredients only involve anthracyclines	59,489
Combined	reports of the active ingredients involve both anthracyclines and dexrazoxane	597
Dexrazoxane alone	reports of the active ingredients only involve dexrazoxane	113

**Table 4:** Conditions for positive signals by disproportionality analysis.

Disproportionality Analysis	Conditions for Positive Signals
ROR ^1^	The number of the reports ≥3 and the lower limit of the 95%CI ^5^ > 1
PRR ^2^	The number of the reports ≥3 and the lower limit of the 95%CI > 1
MHRA ^3^	The number of the reports ≥3 and PRR ≥ 2 and χ^2^ ≥ 4
BCPNN ^4^	The number of the reports ≥3 and the lower limit of the 95%CI > 0

^1^ ROR, reporting odds ratio. ^2^ PRR, the proportional reporting ratio. ^3^ MHRA, the Medications and Health Care Products Regulatory Agency. ^4^ BCPNN, Bayesian confidence propagation neural network. ^5^ CI, confidence interval.

## Data Availability

The original data are available in the FAERS database (https://fis.fda.gov/extensions/FPD-QDE-FAERS/FPD-QDE-FAERS.html) (accessed on 1 June 2024).

## Abbreviations

AE	adverse event
ALL	acute lymphoblastic leukemia
AML	acute myeloid leukemia
ARC	anthracycline-related cardiotoxicity
ATC	Anatomical Therapeutic Chemical
BCPNN	Bayesian confidence propagation neural network
CHMP	Committee for Medicinal Products for Human Use
CI	confidence interval
EDTA	ethylenediaminetetraacetic acid
EMA	European Medicines Agency
EU	European Union
FAERS	Food and Drug Administration Adverse Event Reporting System
FDA	Food and Drug Administration
LVEF	left ventricular ejection fraction
MedDRA	Medical Dictionary for Regulatory Activities
MHRA	Medications and Health Care Products Regulatory Agency
PRR	proportional reporting ratio
PT	preferred terms
ROR	reporting odds ratio
SMN	Second Malignant Neoplasm
SOC	system organ class
US	United States
